# Reaction to Novel Objects and Fecal Glucocorticoid Metabolite Levels in Two Species of Nocturnal Geckos

**DOI:** 10.3390/ani13213384

**Published:** 2023-10-31

**Authors:** Gloria Fernández-Lázaro, Roberto Latorre, Juan Carlos Fontanillas Pérez, Isabel Barja

**Affiliations:** 1 Departamento de Psicología Biológica y de la Salud, Facultad de Psicología, Universidad Autónoma de Madrid, 28049 Madrid, Spain; 2 Departamento de Didáctica de las Ciencias Experimentales, Sociales y Matemáticas, Facultad de Educación, Universidad Complutense de Madrid, 28040 Madrid, Spain; 3 Departamento de Ingeniería Informática, Universidad Autónoma de Madrid, 28049 Madrid, Spain; roberto.latorre@uam.es; 4 Departamento de Fisiología, Facultad de Veterinaria, Universidad Complutense de Madrid, 28040 Madrid, Spain; juancarlos@vet.ucm.es; 5 Eco- and Ethophysiology Lab, Departamento de Biología, Unidad de Zoología, Universidad Autónoma de Madrid, 28049 Madrid, Spain; isabel.barja@uam.es; 6 Centro de Investigación en Biodiversidad y Cambio Global (CIBC-UAM), Universidad Autónoma de Madrid, C. Darwin 2, 28049 Madrid, Spain

**Keywords:** gecko, object novelty, reptiles, animal welfare, individual differences, physiological stress, fecal corticosterone metabolites, nocturnal species

## Abstract

**Simple Summary:**

Reptiles are commonly housed in zoos, wildlife parks or as pets, although there are many species for which welfare measures are not well established. Compared to mammals or birds, they have been under-researched, and, in this study, we focused on two of these species: crested geckos (*Correlophus ciliatus*) and leopard geckos (*Eublepharis macularius*). These geckos are nocturnal, and many questions remain unknown about their behavior or physiology indicators to properly implement welfare protocols. Here, we measured the reaction to novel objects and fecal glucocorticoid metabolite levels, trying to identify any relationship between them. Additionally, we assessed if some characteristic of the objects (e.g., color, shape, or smell) resulted in being more attractive to some species and/or individuals having the potential of being used as enrichment. No preference for any object was found and individuals which manipulated earlier and interacted longer with novel objects showed lower basal fecal glucocorticoid metabolite levels. Crested geckos had significantly greater and more variable fecal glucocorticoid metabolite levels than leopard geckos. These results can help to understand the reaction of geckos to novelty and have the potential to serve in their welfare assessment.

**Abstract:**

Many reptiles are maintained in captivity and heavily traded, although welfare measures for many species are not well established and are under-researched compared to other animals. In this study, we focused on two of these species: crested geckos (*Correlophus ciliatus*) and leopard geckos (*Eublepharis macularius*). To better interpret their behavior in captivity, the individual reaction to novel objects and the fecal glucocorticoid metabolite levels were measured in an attempt to identify the potential correlation between them. Also, we explored if some characteristic of the objects (e.g., color, shape, or smell) resulted in being more attractive to some species and/or individuals. Equivalent responses to different objects were not obtained for all the geckos, the behavioral response being highly individual and context-dependent, although modulated by the species. Individuals which manipulated earlier and interacted longer with novel objects showed lower basal fecal corticosterone metabolite (FCM) levels. Differences according to the species suggested that crested geckos have significantly greater and more variable FCM levels than leopard geckos. Our results can help to understand the reaction of geckos to novelty and have the potential to serve in their welfare assessment, although more studies are needed to proper establish welfare protocols.

## 1. Introduction

The number and species of reptiles housed in captivity is astonishing, and their maintenance has increased in the last 20 years [1]. According to Auliya et al. [2], between 2004–2014, the European Union member states officially reported the import of 20,788,747 live reptiles (CITES and non-CITES species). Other authors also point out that globally 35% of reptile species are traded online, and that three quarters of this trade is in species that are not covered by international trade regulation [3]. All these data pose not only concerns about their conservation status but also for their wellbeing, since many publications confirm the poor knowledge of their welfare compared to mammals or birds, e.g., [4,5,6,7]. Signs of pain or disease are usually less understood, and their behavior has been under-researched, often considered as less behaviorally expressive [8].

Given this situation, not only zoos committed to providing high standards of welfare to their animals but also regulation agencies and veterinary professionals promoting responsible pet care have expressed interest in the last years about research in reptile welfare measures. Examples of this trend reveal the identification of reptile-based welfare indicators for members of the families Agamidae, Chelidae, Pythonidae, Testudinidae [9] and Scincidae [10]. These studies offer measures some of which could be used for other families of reptiles and adapted for species-specific behaviors and requirements. Behavioral and physiological indicators are among the most popular used in welfare assessments, but given the diversity of the order and the lack of information, there is a huge number of species in need of studies and validation of their measures to properly develop and implement welfare protocols. Understanding and identifying, for example, when, how or why reptiles are stressed are important elements for welfare practice, and different authors highlighted the need to further explore non-invasive measures [10,11]. Samples, such as fecal, urinary, salivary or skin, have been already used in some species, e.g., [12,13,14], instead of traditional invasive methods such as blood testing, representing a promising area of research to measure glucocorticoid metabolite levels. Along with this, other authors stated the importance of assessing the welfare of reptiles at an individual level within species, since differences has been found already in responses to novel objects and environments [15]. In fact, curiosity towards novel objects, sounds or smells has been used as a personality assessment tool in many animals, e.g., [16,17], and in general, individuals that are fearful, shy, or not inquisitive will often show an increased latency to approach these new stimulus [18]. However, for reptiles, although many articles have shown evidence of personality in different species, e.g., [19,20,21,22,23,24], there is a need to further explore these inter-individual differences in behavior that are consistent over time and situations along with physiological measures to understand the level of stress experimented on and to properly interpret their reactions along with the implications for their welfare in captivity. For instance, it has been shown that more active Texas horned lizards (*Phrynosoma cornutum*) in their home tanks were also more likely to be explorative in a novel environment, which may require more environment enrichment and more space than that of their less active counterparts [25]. In adult common lizards, more sociable and active personality types had a lower metabolic expenditure, and more aggressive individuals tended to have a higher plasmatic level of corticosterone [26]. In Aldabran giant tortoises (*Aldabrachelys gigantea*), individuals showed variation in preferences when given the choice among different stimuli, demonstrating the importance of considering choice and control [27], and for species with tail autotomy, several authors explored the implications that this behavior can have for their personalities [28], showing, for example, that it is associated with boldness in males but not in females of water anoles [29].

Following these perspectives, in this study we focused on two species of geckos: crested geckos (*Correlophus ciliatus*) and leopard geckos (*Eublepharis macularius*). These species are commonly kept and bred in captivity, making them valuable candidates for research. Consequently, they have been extensively used, especially leopard geckos, as a model species in studies of neurogenesis [30], epimorphic regeneration [31] or color pattern development [32]. Nevertheless, there are not many studies focused on welfare measures for them. Two studies examining the role of enrichment to improve welfare for leopard geckos found that they respond to environmental enrichment [33,34]. Despite differences found in the reaction of geckos to novel stimuli and objects in these studies, both studies agreed on the need to continue exploring these issues, incorporating, for example, levels of physiological stress to validate behavioral measures or evaluations of the preferences of different objects to better develop new enrichment strategies, including species-specific or preferred behavioral types. Similarly, Kundey and Phillips [35] also studied the recognition of novelty in leopard geckos and concluded that they recognized changes in object identities and locations. Moreover, they also suggested to further explore how geckos interact with various types of objects, identifying what domain or domains drive their exploration (e.g., size, shape, brightness, etc.) or how the recognition of novelty affects behavior in situations when escaping from a threat (i.e., a predator). Another investigation on the response of visual stimuli of three species of geckos (*Correlophus ciliatus*, *Eublepharis macularius*, and *Phelsuma laticauda*) found that while geckos responded more often to novel than familiar images the color did not influence the response, suggesting that object content may be more informative than coloration [36]. Studies on other species of geckos explored, for example, the role of personality in their distribution in Australia, finding that while invasive house geckos (*Hemidactylus frenatus*) were not bolder than native house geckos (*Gehyra dubia*), their increased exploratory activity may have contributed to their successful expansion into the natural environments surrounding urbanized areas [37]. With Tokay geckos (*Gekko gecko*), Szabo and Ringler [38] measured neophobia and neophilia and found that individuals hesitated to attack novel prey and prey close to objects (familiar and novel).

With this background, our study aimed to give insights into some of the questions raised by other authors by measuring the reaction to novel objects and the level of fecal glucocorticoid metabolites of leopard and crested geckos, while also exploring the potential correlation between behavioral response and physiological indicators. To identify if some characteristic of the objects (e.g., color, shape, or smell) resulted in being more attractive to some species and/or individuals, we searched for equivalences in the reaction to the novel objects. Specifically, we aimed to observe qualitatively consistent behaviors of every animal when exposed to specific objects. To answer this question, we verified possible equivalences between objects considering the whole population, and also distinguishing between sexes and/or species. Based on the existing literature, we hypothesized that objects that offer opportunities for hiding or climbing might be more attractive than objects that did not [34,35], while scent will elicit equivalent reactions if it is perceived as scent of a predator [39].

To measure glucocorticoid metabolite levels, we decided to use fecal hormonal analysis for its numerous benefits compared to other methods, including being entirely noninvasive, ease of collection and storage, sampling being attainable for smaller species, and reliable assays being commercially available for quantification [13]. We expected corticosterone metabolite levels to be sex- and/or species-dependent [40,41] and related to personality, for example, with individuals who manipulated earlier and interacted longer with novel objects being less fearful and with lower cortisol metabolite levels. The data collected in this work will improve our understanding of gecko reactions to novelty, which could have practical implications for their welfare.

## 2. Materials and Methods

### 2.1. Subjects and Housing

This study involved 17 adult geckos from 2 different species (Table 1): 10 crested geckos (*Correlophus ciliatus*, 9 males and 1 female) and 7 leopard geckos (*Eublepharis macularius*, 3 males and 4 females). All subjects were housed individually at the reptile facility in the Veterinary faculty at the Universidad Complutense de Madrid (Spain). Enclosure measures for the leopard geckos were 40 cm long, 60 cm wide, 40 cm high and for the crested geckos 70 cm long, 40 cm wide and 50 cm high. Temperature oscillated from 26 to 29 °C and humidity was around 40%, although for crested geckos water mist was provided in their terrariums to raise the humidity. The photoperiod was maintained on a 12 h day/night cycle, and lights went off at 18:00 h. All the terrariums had sand substrates and one or two hiding areas with constant water access. For crested geckos, terrariums had branches and vegetation, since this species is arboreal. Diet consisted of insects (crickets and mealworms) and a mix of chopped fruits for crested geckos and only insects for leopards 2/3 times per week, dusted with calcium and vitamins [34,36].

All the procedures were carried out in accordance with the regulations on the protection of animals used for scientific purposes: Directive 2010/63/EU of the European Parliament and of the Council and the Spanish legislation (Royal Decree 53/2013). The project was approved by the Ethical Committee on Animals used in Research of the Faculty of Veterinary at the Universidad Complutense de Madrid (number 08/2018).

### 2.2. Experimental Design

Data collection lasted 4 months, from February to May 2019, during the afternoons (from 17:00 to 20:30 h) to adjust to the active period of the animals. Both the collection of fecal samples and the novel object tests were conducted in the home cages of the geckos. Video and photos in nocturnal conditions were taken with two cameras: video with a Sony handycam hdd hard disk drive nightshot plus DCRA-C162 (Sony Group Corporation, Minato City, Tokyo, Japan) and photos with a Victure HC200 wildlife camera 12 MP with 24 infrared LED lights (Victure, Guangming New District, Shenzhen, Guangdong, China).

#### 2.2.1. Fecal Corticosterone Metabolite Levels

Feces were easily found for each leopard gecko but for the crested geckos were very difficult to spot within the vegetation and branches. Therefore, a blue food-coloring paste (Manuel Riesgo, S.A., Madrid, Spain) was added to the mix of chopped fruits to easily find the feces of this species. This paste is harmless to the animals, and they have been used in many studies with different vertebrate species [42,43]. Only fresh fecal samples were collected to prevent the action of environmental conditions and degradation by microorganisms [44,45]. Individual fecal samples were placed in Ziploc bags and stored in a freezer at −20 °C prior to hormonal analysis. At the end of the study, we had a total of 200 feces, and each animal had between 6 and 17 samples.

For the fecal corticosterone metabolite (FCM) extraction, fecal samples were thawed and dried to a constant weight (90 °C, 5 h). We placed 0.05 g of dry feces in assay tubes with 0.5 mL of phosphate buffer and 0.5 mL of 80% methanol; then, they were shaken for 16 h and 30 min and supernatants were centrifuged at 2500 rpm for 15 min. Pellets were discarded and the fecal extracts were stored at –20 °C until analyzed in the Ethology lab of the Zoology Unit at the Universidad Autónoma de Madrid. We used a commercial corticosterone enzyme immunoassay (DEMEDITEC Diagnostics GmbH, DE-4164 Kiel, Germany) for the quantification. Parallelism, accuracy, and precision tests have to be carried out to validate any enzyme immunoassay [42,44,46]. Parallelism was performed with serial dilutions of fecal extracts (1:32, 1:16, 1:8, 1:4, 1:2, 1:1), resulting in a curve parallel to the standard. Recovery was 109.4%. Precision was tested through intra- and inter-assay coefficients of variation for fecal samples, being 8.4% and 11.4%, respectively. In each assay, we used a standard, whose corticosterone metabolite levels were known, included in the kit. FCM levels were expressed as ng/g dry feces.

#### 2.2.2. Novel Object Test

Each gecko was tested individually in their enclosures with 6 different objects selected according to their characteristics (size, texture, color, reflection and scent). Two little plastic insect figures (one a dragonfly and the other a butterfly, 7 cm long and wings 8 cm wide), two soft bigger figures with different colors (a yellow Pacman toy with a diameter of 6.5 cm and a red heart toy, with 7.5 cm), a mirror (11 cm long and 7.5 cm wide) and a scented wooden block (5 cm long, 3 cm wide). The scented block was created with feces of two snakes (*Boa constrictor* and *Corallus hortulanus*), which can be perceived as potential predators. One teaspoon of both feces was mixed with 5 mL of distilled water and placed in the block, which was air-dried for one hour before use. To control for changes in concentration over time and depositions of the geckos, one scent block was used per animal and then discarded. Each gecko was presented with the novel object in its enclosure, and after 10 min the experimental stimulus was removed. The duration of the test and the objects were selected following previous articles on geckos and other reptiles that use similar items as enrichment or to evaluate personality, e.g., [15,17,25,33,35]. All the objects except for the scented blocks (Figure S1: Photo of novel objects (Butterfly, dragonfly, heart, Pacman and mirror) were disinfected with distilled water and chlorhexidine before being tested with another individual to remove any chemical cue or to be equally unscented. The order of individuals in each test was assigned randomly, but at least 8 days passed before the presentation of a different object to the same gecko. All geckos received the same order of presentation of the object, and they were presented always in the same location in their terrariums. To avoid disturbance of the animals while placing the object and to be able to record their behavior, the position was near the glass at the right or the left of the enclosure (Figures S2−S4).

All the tests were video-recorded, to be coded later using continuous focal sampling on the free behavioral coding software BORIS (Behavioral Observation Research Interactive Software, version 7.13.6 for Windows) [47]. During coding, observers recorded the onset and offset of 8 state behaviors (Table 2 and Videos S1–S8) based on previous ethograms of geckos [33]. For these behavioral categories, we analyzed the latency of approximation (time needed to be in proximity), latency of manipulation and duration, and frequency of all the behaviors. Reliability data were gathered by a second coder for 10% of the tests. The Spearman’s correlation coefficient between the measures obtained by the original and the second coder was 0.997, demonstrating consistency between observers.

### 2.3. Data Analysis

#### 2.3.1. Behavioral Measures

To characterize the different responses of each animal during the tests with novel objects, we used the percentage of the experiment they spent in each of the eight behaviors included in the ethogram (Table 2), as well as the approximation and manipulation latency, also expressed as a percentage of the experiment. Hereafter, we call these values behavioral measures.

When an animal did not approach or manipulate the object throughout the experiment, instead of assigning a score equal to 0%, which would correspond to latencies equal to 0 s, we considered the corresponding latency measure to be equal to 100%. This allowed a continuous linear interpretation of all the behavioral measures in subsequent analyses.

In a first approach, we considered each animal having 10 behavioral measures per novel object. Subsequently, we grouped outcomes of different experiments, i.e., observations of different novel objects, into a single set of behavioral measures by computing the median of every measure across all the experiments being combined. The criteria used to group novel object tests are presented in the results section.

#### 2.3.2. FCM Score

In the case of the corticosterone metabolite levels, to obtain a single characteristic FCM score for each gecko, we used the mean FCM concentration of all feces collected for the corresponding subject. Samples with a FCM concentration below 0.01 ng/g were discarded for this calculation.

#### 2.3.3. Correlation and Principal Component Analyses

We looked for possible linear correlations among the different behavioral responses observed during the novel object tests, and also between the behavioral measures and the FCM level by means of Spearman’s rank correlation (ρ). We performed individual and group analyses according to sex and species. However, due to the small number of individuals in our test group, meaningful group comparisons according to sex and species together were not possible.

These analyses allowed us to identify (i) equivalences between novel objects and (ii) possible relationships between behavior and FCM levels. In the linear correlation analyses, only the Spearman coefficients with a confidence level above 95% were considered statistically representative. In relevant cases with enough significant statistics, paired comparisons across attributes were assessed by the Wilcoxon–Mann–Whitney (WMW) test.

To find more complex relationships in the behavioral responses of the geckos and group them as a function of their characteristic behavior during the novel object tests, we used the K-Means clustering algorithm. To reduce the dimensionality in these analyses and/or identify the main sources of variability of our datasets with independent variables, we performed a principal component analysis (PCA).

Finally, we tried to correlate the possible responses identified during the K-Means analysis with the characteristic FMC level of each animal. All statistical analyses were performed in MATLAB R2023a for Linux.

## 3. Results

### 3.1. Equivalence of Novel Object Tests

**Figure 1:**
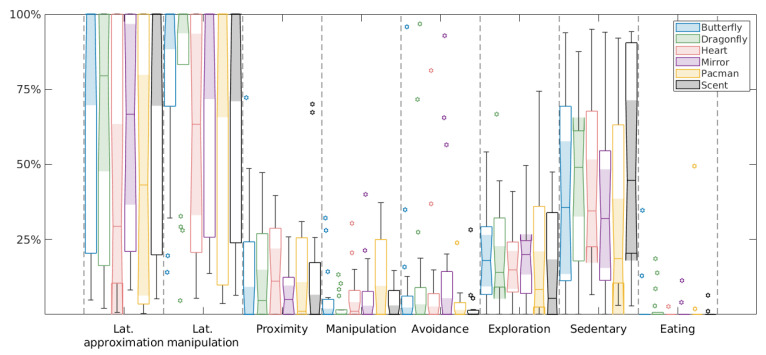
Boxplot illustrating the variability of the behavioral response of all the geckos to each novel object.

A first noteworthy observation in our experiments was a high response variability, both between animals and between experiments involving the same animal (Figure 1). Among others, this variability meant that equivalent responses to different objects were only obtained for specific behaviors and not for the entire ethogram. Thus, in the whole tested group (17 subjects), we did not find any behavioral measure that could be considered statistically equivalent for two different novel objects. In the Pacman and heart experiments, all the leopard geckos approached the object except for L1, but some individuals while approaching did not manipulate them, as did L4. For crested geckos, the variation was even bigger, with C2, C3 and C4 not approaching them, and some individuals approaching and manipulating them and some not. The same tendency resulted with the butterfly and the dragonfly, with individuals of both species not approaching them, some approaching and not manipulating and other even manipulating them.

Similarly, during the mirror experiment, three males (subjects C7, L5 and L2) approached the object more than once and accordingly spent 40%, 21% and 19% of the time in manipulating it. By contrast, subjects L3, L7, L1, C3, C4, C6 and C8 did not approach or manipulate the mirror.

Finally, in the scented wooden block experiment, all the leopard geckos except for L6 approached and manipulated the block while crested geckos mostly showed sedentary behavior (76% of the experiment in mean) and did not manipulate it, except for C8, which spent 4% of the experiment manipulating it.

For males (12 subjects), every animal showed an equivalent avoidance response in the experiments with the scented wooden block and in the experiments with the butterfly; an equivalent latency of manipulation with the Pacman and the dragonfly; and an equivalent eating response with the scented wooden block and the Pacman. In the case of females (five subjects), the experiments with the dragonfly and the butterfly seemed to be equivalent in terms of latency of approximation and manipulation, proximity and manipulation response; while experiments with the scented wooden block and the Pacman seemed so in terms of avoidance response. Furthermore, in some sense, the females’ latency of manipulation in the experiments with the heart was related to the latency of manipulation in the experiments with the Pacman, since those that took longer to manipulate the heart did not manipulate the Pacman and vice versa.

In summary, we did not find common responses as a function of some characteristic of the object. Consequently, to characterize the behavioral response of our geckos, we used the same approach as in [42], and combined the results obtained with different objects into a single set of behavioral measures characterizing the average response of each animal, rather than the response to specific objects.

### 3.2. Individual and Group Response to All Novel Objects

**Figure 2:**
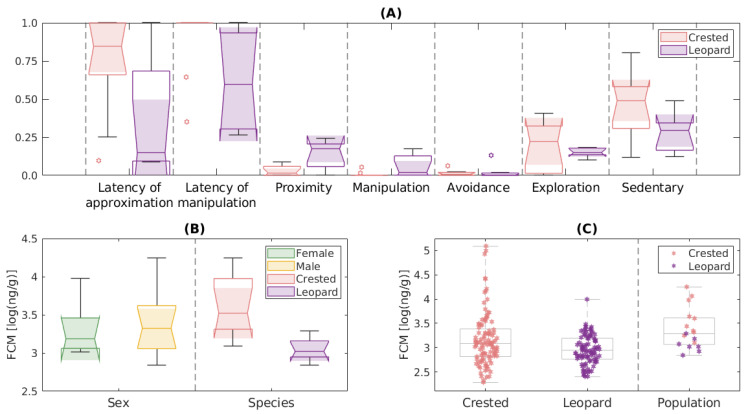
(**A**) Boxplot characterizing and comparing the behavioral response of crested and leopard geckos in the novel object tests. (**B**) Characterization of FCM levels of all geckos divided by sex (males or females) and by species (crested or leopard). (**C**) The FCM level of each individual grouped by species.

Looking for possible explanations in the response between sexes, we found that the behavioral categories ARB, eating and “other” were mainly observed in males of both species. Nevertheless, they only appeared occasionally in a limited number of experiments—cf. the eating response in Figure 1, where the animals that ate during the experiments are considered statistical outliers as compared with the ones that did not eat— making it impossible to correlate the occurrence of any of these behaviors with a given sex. Indeed, the lack of statistics on these behaviors was a general outcome in all the group comparisons performed, pointing to their individual and context dependency. Focusing on the rest of behavioral dimensions analyzed, the behavior of males and females was so variable that no sex-dependent responses were found. In contrast, we did find some statistically significant differences between species. Figure 2A shows a boxplot illustrating and comparing the responses of leopard and crested geckos. Our average leopard gecko manipulated the object earlier and interacted longer with it than the average crested gecko (latency of manipulation = 0.60 ± 0.32 vs. 0.90 ± 0.22, WMW test p-value = 0.035; time in proximity = 0.03 ± 0.03 vs. 0.14 ± 0.09, WMW test p-value = 0.019; and manipulation time = 0.06 ± 0.07 vs. 0.01 ± 0.02, WMW test *p*-value = 0.035). No other statistically significant difference between species was found.

Correlation analysis among the characteristic behavioral measures of each animal revealed some significant general relationships (see Table 3). First, we found a positive correlation between latencies of approximation and manipulation, in such a way that the animals that approached the novel object more quickly also took less time to start manipulating it, while those that took longer to approach the object (and obviously the ones that did not approach) did not manipulate it. Both latencies correlated negatively with the proximity and the manipulation response of the animal. Thus, the individuals that spent the longest time interacting with the object—i.e., manipulating it or in its proximity—were the ones that had approached it the fastest.

Second, there was a general moderate–high correlation between the four previous behavioral measures and the sedentary response of the animal. The correlation was positive in the case of the approximation and manipulation latency and negative for the proximity and manipulation behavior. Thus, the greater the sedentary response of an animal, the longer it took to approach and manipulate the object and the less time it spent interacting with it. In this regard, it also important to note that the animals that did not interact with the object at all were the most sedentary.

Finally, as could be expected, exploration and sedentary were negatively correlated, but no other significant relationship was found.

### 3.3. Classification of Individual Behavioral Responses

A PCA analysis on the individual behavioral measures indicated that the two first principal components explained more than 91% of the total variability of our geckos’ response during the novel object tests. Table 4 shows the percentage of variability that each of these PCA components explained, together with their coefficients. These coefficients suggested that the more relevant variables to characterize and classify the different behaviors observed in our experiments—those with the highest coefficients in absolute value—were latencies of approximation and manipulation and exploratory response. Taking this dimensionality reduction into account, we performed a K-means clustering analysis to classify the different responses as a function of these three variables. In this way, we identified an optimal classification dividing our gecko population into four representative groups. Column “Behavioral group” of Table 1 indicates the group to which each subject belonged. The centroids characterizing the response of the four identified groups are shown in Table 5:

- Group B1 included animals that did not approach or manipulate the object, nor did they display exploratory behavior.
- Group B2 included animals with a significant exploratory behavior that took time to detect and approach the object, but they did not manipulate it.
- Group B3 included animals with a significant exploratory behavior, initially attracted by the object although they needed time to manipulate it.
- Group B4 included animals attracted to the object from the beginning of the experiment that “frequently” manipulated it or stayed close to it.

Apart from group B3, which contained a single animal, as in previous analyses, none of these behaviors could be exclusively linked to a particular sex or species, although some general trends could be identified. It appeared that the response of the animals in group B1 was typical of crested geckos, while the response of animals in group B4 was that of leopard geckos. In contrast, group B2 contained animals of both species, although interestingly all the crested geckos were male, and all the leopard geckos were female, suggesting that some males of one species behaved like females of the other species and vice versa. However, this last result is subject to further validation because in our study we only had a female crested gecko. Finally, subject C1, the only subject included in B3, from a behavioral perspective, in some sense could be considered an outlier since it displayed a response completely different to the rest of individuals. Accordingly, the K-means algorithm created a cluster exclusively for it.

### 3.4. Corticosterone Metabolite Levels

We started our analysis by trying to find differences in the characteristic FCM level of different sexes and species (Figure 2B). Although fecal samples from males in general had a higher average FCM levels than feces from females (4188 ± 5182 ng/g vs. 3029 ± 3614 ng/g), as in the case of the behavioral response, there was so much variability among individuals that no statistically significant differences were found between sexes. In the comparison between species, if we exclusively focused on the average FCM level of each animal, at a first glance it seemed that each species had a different basal FCM level, with crested gecko showing significantly greater FCM levels than leopard gecko (5714 ± 5444 ng/g vs. 1181 ± 424 ng/g, WMW test *p*-value = 0.001). However, as observed in the left panel of Figure 2C, we should interpret these results carefully, because for crested geckos some samples had very high FCM levels compared to all other collected samples. What seemed clear was that the animals with the highest average FCM levels were crested geckos, while those with the lowest values were leopard geckos, and that in the intermediate concentrations individuals of both species appeared mixed.

The observed dependence of the FCM levels on the species led to some moderate correlations between the FCM levels and those behaviors that were species-dependent, which allowed us to describe some general tendencies. Thus, we found positive correlations with the latency of approximation (ρ = 0.66, *p* = 0.004) and the latency of manipulation (ρ = 0.50, *p* = 0.041); and a negative correlation with the proximity response (ρ = −0.66, *p* = 0.004). Additionally, we analyzed the relationship among the FCM levels and the main PCA components of the individual behavioral response, finding a moderate positive correlation with the first PCA component (ρ = 0.63, *p* = 0.008). Although they allowed describing some general trends, none of the observed correlations could by themselves explain the individual FCM levels of the geckos.

**Figure 3:**
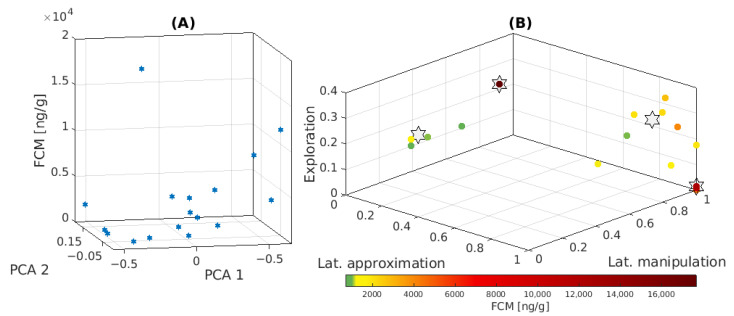
(**A**) Correlation of the two main PCA components of the behavioral response with the individual FCM level. (**B**) Correlation between the FCM level of each individual with the three behavioral measures that the PCA analysis identified as the most relevant: latency of approximation, latency of manipulation and exploration. Hot colors correspond to individuals with the greater FCM levels, cold colors to individuals with the lower ones. The centroids of the four clusters identified by K-means analysis appear as stars.

We also analyzed the combined correlation of the two main PCA components of the behavioral response with the individual FCM level (see Figure 3A). Neither the second PCA component (ρ = 0.37, *p* = 0.147) nor its most significant term, i.e., the exploratory response (ρ = 0.07, *p* = 0.779), seemed to be correlated with the FCM levels. However, considering both PCA components together allowed identifying more complex relationships. For the sake of a better understanding, Figure 3B confronts the characteristic FCM levels of each individual with the three behavioral measures that the PCA analysis identified as the most relevant to classify the geckos as a function of their behavior. The gray stars in this figure represent the centroids of the four clusters identified by K-means as the optimal behavioral classification of the animals. Comparing the FCM levels and this behavioral classification, there seemed to be some correlation between them, so that animals with similar behavior seemed to show similar FCM levels. Thus, the animals that showed exploratory behavior and were attracted to the object from the beginning of the experiment and frequently manipulated it or remained close to it (Group B4), in general, had low FCM levels (as compared with the rest of the individuals of the same species). At the other extreme were the animals that did not approach or manipulate the object and did not show exploratory behavior either (Group B1). These showed high FCM levels. In between these two groups, the animals that explored the environment but were slow to approach and manipulate the object (Group B2) showed intermediate FCM levels. Finally, the animal with an extremely high FCM level was classified alone by K-means in Group B3.

## 4. Discussion

To our knowledge, this is the first study that analyzes reaction to novel objects in crested and leopard geckos, trying first to detect if some characteristic of the objects resulted in their being more attractive to some species and/or individuals. However, results of our tests did not depend on any shared feature of the object. For example, we expected that the objects that offer opportunities for hiding or climbing (our soft bigger figures: Pacman and heart) might be more attractive to geckos [34,35], and this was not the case. We saw that some individuals rapidly approach and manipulate the soft bigger figures, climbing them (Figures S5 and S6: individuals manipulating and climbing the soft bigger figures (Pacman and heart)), while others did not, and they did not differ in duration of manipulation or proximity compared with other novel objects. A possible explanation for these different results could be that the objects used in our experiments were quite different from those used in previous work. For example, Zieliński [34] used cork oak bark in his dry hide treatment, and Bashaw et al. [33] used branched sticks or a flexible wood bridge placed under the heat lamp as thermal enrichment for leopard geckos. Nevertheless, we could not use similar items in the novel object tests, since the shelters offered in the home terrariums of our geckos were already very similar. Our previous assumption that maybe our soft bigger figures could have been seen as possible items to climb was not correct, as most of our geckos showed no differences in their responses to the others object used. Something similar occurred with the mirror, because, as obtained by Bashaw et al. [33], our geckos did not interact significantly longer with the mirror compared with the rest of the objects. Instead, Hall et al. [17] found that Indian star tortoises (*Geochelone elegans*) apparently showed a preference for the mirror in comparison with other objects, even attracting other turtles from the enclosure when one approached it. Nevertheless, these results must be interpreted cautiously, since in that study only three out of five turtles approached and manipulated the mirror. Also, it has been shown [48] that for some tortoises (*Testudo hermanni*) the mirror can act as a social reinforcement with a lateralized social behavior. But, from our results, we cannot know if the reflection can be perceived as a social stimulus or as a competitor by both crested or leopard geckos, and it would be interesting to explore these issues with bigger samples.

The only test that in our study showed some differences by species was the scent. Leopard geckos approached and spent in proximity between 11% and 67% of the experiment, even manipulating it, while eight of our crested geckos did not approach it. This result could be interpreted as the different antipredation strategies that both species had, because as Landova et al. [39] found out, leopard geckos explored snake predators chemically and visually, reacting with intensity to boid snakes (the feces used in this study), with captive-born individuals being more reactive. It is true that the boid species used here are not natural predators of them, and that is why the predator inspection showed in our leopards or the reduced activity in the presence of the scent in crested geckos, passing between 41% and 91% of the experiment in sedentary behavior, could have been caused by uncertainty of the risk assessment. According to the threat-sensitivity hypothesis, animals should adjust their anti-predator responses according to the level of threat posed by specific predators, i.e., [49,50]. For example, it has been shown that Asian house geckos (*Hemidactylus frenatus*) did not respond to the chemical cues of all snakes in the same way [51] or that scincid lizards (*Carlia rostralis*, *C. rubrigularis* and *C. storri*) selectively avoid chemical cues from dangerous varanid predators and ignore those of the less dangerous [52]. Therefore, if our geckos could not distinguish the level of threat or even if the scent represented a threat at all, this could cause the different responses. Also, in contrast to Landova et al. [39], which presented the snake, we only presented the chemical cues and in a different manner to the one used in other studies with scented filtered papers [52,53,54]. However, as Cornelis et al. [51] pointed out, it is unclear which method is more effective in capturing appropriate predator odors or which specific chemicals induce the antipredator behavior, and more studies in this area are needed to properly interpret these differences.

Overall, we did not find repeatability among pairs of novel objects tested, but when we combined all of them, we found individual repeatability among behavioral measures (latencies of approximation and manipulation, duration of proximity, manipulation and sedentary behaviors) as was previously obtained for different behavioral traits in other reptiles, confirming the presence of what we called personality [20,55,56,57]. For example, repeatability of behavioral traits of activity, aggressiveness and risk taking was shown in common lizards (*Zootoca vivipara*) [26] or in headstart Texas horned lizards (*Phrynosoma cornutum*) for behavioral responses of activity and exploration [25]. In fact, in this last study lizards that were more active in their home tanks were also more likely to be explorative in a novel environment, which is similar to what we saw, since the greater the sedentary response of our geckos, the longer it took an individual to approach and manipulate the object and the less the time spent interacting with it. However, the exploration behavior was not related to the reaction to novel objects and was negatively correlated with the sedentary behavior in our geckos. We thought that this is because what we label as “exploratory behavior” represents the active–sedentary traits, and there is a difference between exploratory–avoidance and shyness–boldness. In fact, in a metanalysis of studies which used the novel object test, Takola et al. [16] highlight the necessity to clearly establish a label for the behavioral response observed and what kind of test must be developed to avoid ambiguity when comparing results. That is one of the reasons why we decided to use the term “response to novel objects” first and then discuss the possible interpretations and why we added a physiological measure to have a better understanding of the response observed. Given our experimental design of placing a novel object in a familiar environment, we thought that responses are better interpreted as a measure of shyness–boldness (tendency to engage in risky behavior) than exploration–avoidance, which was often measured in open field or novel-environment trials [58]. In fact, our results follow previous findings in other species of geckos, suggesting that exploratory activity and boldness were not interchangeable and represented two different traits [37]. Therefore, we saw that bolder geckos (individuals which interact sooner and manipulated more the objects) were also the less sedentary, which can be related to be being more proactive. Moreover, when we added the FCM levels we saw that these geckos are mainly leopards, which not only manipulated earlier and interacted longer with novel objects than the average crested geckos, but they also showed lower basal FCM levels and less variability. This result is in line with what other authors have shown in identifying bolder or proactive-behavioral individuals with lower levels of physiological stress than shy or reactive ones [59,60,61].

An aspect that has been shown to affect personality in reptiles [28,29], and with a high incidence of presence within the family Gekkonidae [62], is caudal autotomy. In our study, although two crested geckos lost their tails (C1 at the beginning of the study and C2 before the study was started) we could not obtain enough fecal or behavior samples before the losses occurred to compare. However, it is noteworthy that the K-means classified their behaviors in two different groups—in fact, creating one only for C1. If we compare them, we saw that C2 was included in B1 with most of the crested geckos where there are individuals that did not approach or manipulate the object, and C1 was in B3 because it was initially attracted by the object, although it needed time to manipulate it. This result could be linked to what was seen before in water anoles (*Anolis aquaticus*) where males that had experienced tail autotomy were bolder than males that had not [29], and the behavior of C2, since the voluntary loss of the tail was earlier, suggests that the effects could have been normalized. In contrast, Michelangeli et al. [28] found that lizards (*Lampropholis delicata*) with complete tail loss, on average, became less active and explorative, although no effect was found between tail autotomy and the time the lizards spent in a novel food zone. In other species of geckos, some authors find that autotomized geckos (Cape Dwarf Gecko, *Lygodactylus capensis*) were significantly slower than intact geckos during vertical escape [63], *Ch. marmoratus* geckos were faster in escape over horizontal surfaces after loss of their tail [64], and that for frog-eyed geckos (*Teratoscincus scincus*) the antipredator behavior was not modified following autotomy [65]. If we add the FCM levels to our results, we saw also a huge difference, C1 being the individual with extremely high FCM levels and C2 with the lowest mean concentration within the crested geckos. These levels contradict what was before detected in scincid lizards (*Eulamprus heatwolei*), where females who recently were forced to lose their tail had plasma corticosterone levels that increased 1 h after tail autotomy but fell back to control levels within 2 h and remained low 14 days later [41]. Adding to this controversy is the fact that both individuals presented some abnormal repetitive behaviors (only in two sessions and not very long: C1 with a mean value of 22% and C2 of 2% of the experiment). Since performing these behaviors has been shown to liberate stress [66], we can suggest that tail autotomy in these two individuals could be linked to this strategy and that C2, since it performed it before, managed to lower FCM levels, but perhaps C1 is in the process to doing so. All these data pose interesting questions about the effect that caudal autotomy may have in the behavior and at the physiological level for crested geckos and other reptiles, and we encourage future studies to look at it.

Sex differences in behavioral traits were not found as previously seen for other species of geckos [37], although they were detected in other reptiles [67,68]. However, a recent meta-analysis study found no evidence for widespread sex differences in variability in non-human animal personality [69], which is in line with what we saw. Something similar was obtained for the stress response with no significant differences between FCM levels of males and females, in contrast to what we previously thought, since in other reptiles males show higher corticosterone levels than females [12,40,70] or the opposite with females showing higher levels [41]. What we detected were differences according to the species, with crested geckos showing significantly greater FCM levels than leopard geckos. However, given our small sample and that glucocorticoid levels in reptiles can vary between individuals, geographical location, energetic status, reproductive status, age and body condition [11,71,72], we must interpret these results cautiously, since as previously stated some fecal samples showed very high cortisol levels compared to all other collected samples (Figure 2C).

Finally, to limit the scope of the study and to explain its utility, it is important to repeat that we are presenting results based on a small number of individuals. Despite this limitation, our work addresses interesting aspects about the reaction to novel objects, individual behavior and FCM levels in geckos while also identifying future directions of study, concerning, for example, the use and detection of chemical odors, reflections in mirrors or caudal autotomy with bigger samples. It also provides an example to further explore non-invasive measurement methods of stress to better interpret results and to develop protocols which can help to ensure the welfare of these species commonly maintained in captivity.

## 5. Conclusions

Our work suggests that there is a relation between consistent individual differences in behavior toward novel objects and physiological indicators in geckos. Bolder individuals which manipulated earlier and interacted longer with novel objects showed lower basal FCM levels. Differences according to the species suggest that crested geckos have significantly greater and more variable FCM levels than leopard geckos. Although more studies are needed to better understand the connection between personality and the physiological response in geckos, we think that data presented here can be added to the growing literature which seeks to promote the welfare of reptiles in captivity.

## Figures and Tables

**Table 1:** Individual profiles.

Subject	Species	Sex ^a^	FCM ^b^ (ng/g)	Behavioral Group
C1	*Correlophus ciliatus*	M	17,666.4	B3
C2	*Correlophus ciliatus*	M	1239.1	B1
C3	*Correlophus ciliatus*	M	11,631.1	B1
C4	*Correlophus ciliatus*	M	4357.9	B1
C5	*Correlophus ciliatus*	M	2046.7	B2
C6	*Correlophus ciliatus*	F	9464.1	B1
C7	*Correlophus ciliatus*	M	2758.1	B2
C8	*Correlophus ciliatus*	M	3996.7	B2
C9	*Correlophus ciliatus*	M	1791.1	B4
C10	*Correlophus ciliatus*	M	2189.9	B2
L1	*Eublepharis macularius*	F	1931.5	B1
L2	*Eublepharis macularius*	M	843.7	B4
L3	*Eublepharis macularius*	F	1189.1	B2
L4	*Eublepharis macularius*	M	692.0	B4
L5	*Eublepharis macularius*	M	1047.8	B4
L6	*Eublepharis macularius*	F	1030.6	B2
L7	*Eublepharis macularius*	F	1530.7	B4

^a^ M = Male, F = Female; ^b^ Mean value for all the feces collected per individual.

**Table 2:** Behavioral categories used for analyses.

Behavior	Definition
Exploration	Moving around enclosure or climbing vertical surfaces at slow speed in no fixed pattern away from the novel object. It can include tongue flicking.
Sedentary behavior	Lying or standing with eyes open or closed, not engaged in any active behavior.
Proximity	Being within a 2 cm range of the object with any part of the body (at least 50% of it), but the attention is not necessary directed to the object. It can be exploring or sedentary.
Manipulation	Stepping on, climbing, biting, or licking the object but actively paying attention at it. Since geckos can have periods of inactivity followed by quick movements, ten seconds was selected as the cut-off time, as in previous studies, to ensure that the lack of movement was not just a momentary stop [36]. Therefore, if the gecko did not move for more than ten seconds it was considered as Proximity.
Avoidance	Moving or jumping rapidly away from a stimulus and or hiding with face not visible to observer.
Eating	Licking the mix of chopped fruits from the feeder or drinking water.
Abnormal Repetitive Behavior (ARB)	Moving around the enclosure or climbing walls on a set path (at least two repetitions required) or repeatedly attempting to climb out of the cage, scratch or burrow into the glass for more than 20 s.
Other	To scratch the sand or try to bury the novel object.

**Table 3:** Spearman correlation coefficient between behavioral measures. Note that eating, ARB and other were rarely produced behaviors and, therefore, they showed no correlation with any other behavioral measure. Coefficients with a confidence level above 95% are highlighted in bold face.

	Latency of Manipulation	Proximity	Manipulation	Exploration	Sedentary	Avoidance
Latency of approximation	**0.83 (*p* < 0.001)**	**−0.90 (*p* < 0.001)**	**−0.77 (*p* < 0.001)**	−0.43 (*p* = 0.083)	**0.77 (*p* < 0.001)**	−0.12 (*p* = 0.642)
Latency of manipulation		**−0.85 (*p* < 0.001)**	**−0.98 (*p* < 0.001)**	−0.25 (*p* = 0.320)	**0.76 (*p* < 0.001)**	−0.01 (*p* = 0.977)
Proximity			**0.85 (*p* < 0.001)**	0.33 (*p* = 0.197)	**−0.80 (*p* < 0.001)**	0.12 (*p* = 0.635)
Manipulation				0.25 (*p* = 0.326)	**−0.80 (*p* < 0.001)**	0.03 (*p* = 0.923)
Exploration					**−0.53 (*p* = 0.028)**	0.32 (*p* = 0.213)
Sedentary						−0.24 (*p* = 0.350)

**Table 4:** Coefficients of the first two PCA components of the individual characteristic behavioral measures (Coef 1 and Coef 2). The coefficients in boldface correspond to the variables with a greater weight in each component. The column “Variability” shows the percentage of variability explained by the corresponding PCA component.

	Latency of Approximation	Latency of Manipulation	Proximity	Manipulation	Exploration	Sedentary	Avoidance	Eating	ARB	Other	Variability
Coef 1	**0.6766**	**0.5499**	−0.1618	−0.1113	−0.1636	0.4153	0.0400	0.0174	−0.0095	−0.0058	83.0%
Coef 2	0.0931	0.2005	−0.2258	−0.1001	**0.8509**	−0.1595	−0.3731	−0.0353	0.0100	−0.0089	8.7%

**Table 5:** Centroids characterizing the four responses identified by K-Means.

	Latency of Approximation	Latency of Manipulation	Exploration
B1	1.00	1.00	0.01
B2	0.76	1.00	0.22
B3	0.25	0.65	0.34
B4	0.10	0.33	0.18

## Data Availability

Data supporting results of this study can be found in the supplementary materials.

## Supplementary Materials

The following supporting information can be downloaded at: https://www.mdpi.com/article/10.3390/ani13213384/s1, Figure S1: Photo of novel objects (Butterfly, dragonfly, heart, Pacman and mirror); Figure S2: Crested or leopard geckos with novel objects at the right or left of the enclosures – C5 with mirror; Figure S3: Crested or leopard geckos with novel objects at the right or left of the enclosures – C8 with heart; Figure S4: Crested or leopard geckos with novel objects at the right or left of the enclosures – L1 with butterfly; Figure S5: individuals manipulating and climbing the soft bigger figures (Pacman and heart) – L3 climbing heart; Figure S6: individuals manipulating and climbing the soft bigger figures (Pacman and heart) – L6 inside pacman; Video S1: ARB C1 with butterfly; Video S2: Avoidance L3 with scented wooden block; Video S3: Eating or drinking L1 with Pacman; Video S4: Exploration behavior C1; Video S5: Manipulation behavior L6 with Pacman; Video S6: Other L4 with scented wooden block; Video S7: Proximity behavior L5 with dragonfly; Video S8: Sedentary behavior L6 with Pacman; Table S1: Data used in this study; Document S1: Dataset with individual representative data of each gecko.
