# Supplementary material for: Reaction to Novel Objects and Fecal Glucocorticoid Metabolite Levels in Two Species of Nocturnal Geckos
Source: Animals (Basel). 2023 Oct 31;13(21):3384. doi: 10.3390/ani13213384 (PMC10649715; doi:10.3390/ani13213384)
Supplement: Supplementary file 1 [file animals-13-03384-s001.zip › S5/Info Dataset.pdf]

Dataset with individual representative data of each gecko:

- Subject
- Sex
- Species
- FCM: Mean FCM level for the feces collected per individual (ng/g)
- L_Approximation: Latency of approximation (percentage of the experiment)
- L_Manipulation: Latency of manipulation (percentage of the experiment)
- Proximity: Percentage of the experiment exhibiting proximity behavior
- Manipulation: Percentage of the experiment exhibiting manipulation behavior
- Exploration: Percentage of the experiment exhibiting exploration behavior
- ARB: Percentage of the experiment exhibiting abnormal repetitive behavior
- Sedentary: Percentage of the experiment exhibiting sedentary behavior
- Avoidance: Percentage of the experiment exhibiting proximity behavior
- Eating: Percentage of the experiment exhibiting avoidance behavior
- Other: Percentage of the experiment exhibiting other behavior
